# Book Reviews

**Published:** 1846-06

**Authors:** 


*Illinois Medical and Surgical Journal.*—This periodical has been enlarged. It is now published every other month, simultaneously in Chicago, Ill. and Indianapolis, Ind. Each No. contains ninety-six pages, we have received the number for April and May, which contains some very interesting articles. It is under the editorial management of Drs. Blaney, Brainard, Herrick and Evans.—*Balt. Ed.*

*Western Lancet.*—The May Number of the Western Lancet has come to us in an enlarged form. Hereafter it will be published, bi-monthly, each number containing 132 pages. It is still under the able editorial management, of Prof. Lawson, and is published at Lexington, Ky.—*Balt. Ed.*

*An Essay on the Pathology and Treatment of Trismus Nascentium, or Lock-Jaw of Infants.* By J. MARION SIMS, M. D., of Montgomery, Ala., pp. 21, 8vo. Philadelphia: Lea & Blanchard, 1846.

The above is a well written and highly interesting pamphlet. It contains some very novel and ingenious views with regard to the pathology and treatment of a very fatal, and, in some parts of the country, quite a common disease, and we regret that we did not receive it in time to give an analysis of the peculiar opinions of the author on the subject As a surgeon and physician, Dr. Sims enjoys a justly deserved high reputation, and we take this occasion to thank him for the above named essay. The perusal of it has given us much pleasure.—*Balt. Ed.*

*Catalogue of the Trustees, Officers and Students of the Indiana Medical College, Session* 1845–6.

The Indiana Medical College is in Laporte, and appears, from the above catalogue, to be in a very flourishing condition. It has six professorships. The Faculty is composed of Drs. G. W. Richards, John B. Niles, Moses L. Knapp, Daniel Meeker, A. B. Shipman and Nicholas Hard. There were eighty students in attendance upon the lectures of the session of '45–6.

